# Modulation of macrophage transcript and secretion profiles by *Sargassum Serratifolium* extract is associated with the suppression of muscle atrophy

**DOI:** 10.1038/s41598-024-63146-0

**Published:** 2024-06-10

**Authors:** Heeyeon Ryu, Hyeon Hak Jeong, Myeong-Jin Kim, Seungjun Lee, Won-Kyo Jung, Bonggi Lee

**Affiliations:** 1 Department of Food Science and Nutrition, Pukyong National University, 599-1, Daeyeondong, Nam-Gu, Busan, 48513 Republic of Korea; 2 Department of Smart Green Technology Engineering, Pukyong National University, Busan, 48513 Republic of Korea; 3 Division of Biomedical Engineering and Research Center for Marine Integrated Bionics Technology, Pukyong National University, Busan, 48513 Korea; 4 Marine Integrated Biomedical Technology Center, The National Key Research Institutes, PukyongNationalUniversity, Busan, 48513 Republic of Korea

**Keywords:** Macrophages, Skeletal muscle, Cytokine, RNA Seq, *Sargassum Serratifolium*, Cell biology, Immunology, Molecular biology

## Abstract

Recent research has emphasized the role of macrophage-secreted factors on skeletal muscle metabolism. We studied *Sargassum Serratifolium* ethanol extract (ESS) in countering lipopolysaccharide (LPS)-induced changes in the macrophage transcriptome and their impact on skeletal muscle. Macrophage-conditioned medium (MCM) from LPS-treated macrophages (LPS-MCM) and ESS-treated macrophages (ESS-MCM) affected C2C12 myotube cells. LPS-MCM upregulated muscle atrophy genes and reduced glucose uptake, while ESS-MCM reversed these effects. RNA sequencing revealed changes in the immune system and cytokine transport pathways in ESS-treated macrophages. Protein analysis in ESS-MCM showed reduced levels of key muscle atrophy-related proteins, TNF-α, IL-6, IL-1, and GDF-15. These proteins play crucial roles in muscle function. These findings highlight the intricate relationship between the macrophage transcriptome and their secreted factors in either impairing or enhancing skeletal muscle function. ESS treatment has the potential to reduce macrophage-derived cytokines, preserving skeletal muscle function.

## Introduction

Macrophages, versatile sentinels of the immune system, play a pivotal role in maintaining tissue homeostasis and bolstering the fortress of immune defense^1,2^. However, it is essential to recognize that under specific pathological conditions, macrophages may undergo aberrant activation, triggering a cascade of events that lead to the establishment of chronic inflammation^3^. This persistent state of inflammation is unequivocally associated with various health complications, including but not limited to muscular atrophy and disruptions in glucose metabolism^3–6^.

*Sargassum serratifolium*, a distinguished member of the brown algae family, has emerged as a subject of considerable research interest, primarily owing to its commendable anti-inflammatory attributes and its propensity to regulate metabolic processes^7,8^. Derived from *S. serratifolium*, extracts have, unequivocally, demonstrated their prowess in restraining inflammatory cytokines^7^, manifesting antioxidant properties efficaciously scavenging reactive oxygen species, and fortifying cellular fortresses, thereby substantiating their mettle as potent anti-inflammatory agents^8^. Additionally, these extracts have exhibited the propensity to modulate immune responses, offering therapeutic potential in a diverse spectrum of physiological contexts, including but not limited to the amelioration of obesity, non-alcoholic fatty liver disease^9^, attenuation of inflammation, and mitigation of oxidative stress. Furthermore, explorations have unveiled the extracts' potential antimicrobial activity against pathogenic bacteria associated with conditions such as acne and cutaneous candidiasis^10^.

It is pertinent to underscore that muscle wasting, characterized by the atrophy of muscle mass and the concomitant loss of functionality, may be construed as a consequential outcome of chronic inflammation, and is unequivocally associated with elevated morbidity and a diminished quality of life across a spectrum of maladies^11,12^. In recent years, emerging evidence has unequivocally underscored the central role of macrophages in the regulation of immune responses governing muscular homeostasis and atrophy^13^. The dynamic recruitment of eosinophils to damaged or affected muscle tissues, exhibiting phenotypic plasticity transitioning from inflammatory (M1) to anti-inflammatory and regenerative (M2) states, orchestrates the secretion of factors that profoundly influence the fate and functionality of neighboring muscle cells^14,15^.

Our study primarily aims to explore how the ethanolic extract of *S. Serratifolium* (ESS) modulates macrophage transcriptome and secretion factors, and how these changes ultimately influence muscle atrophy. Specifically, we investigated how macrophage secretion factors, influenced by either LPS treatment alone or LPS combined with ESS treatment, could impact muscle atrophy signaling using RAW 264.7 and C2C12 cells.

## Results

### Impact of ESS-MCM on muscle atrophy, inflammatory response, and glucose uptake in C2C12 myotubes

**Figure 1:**
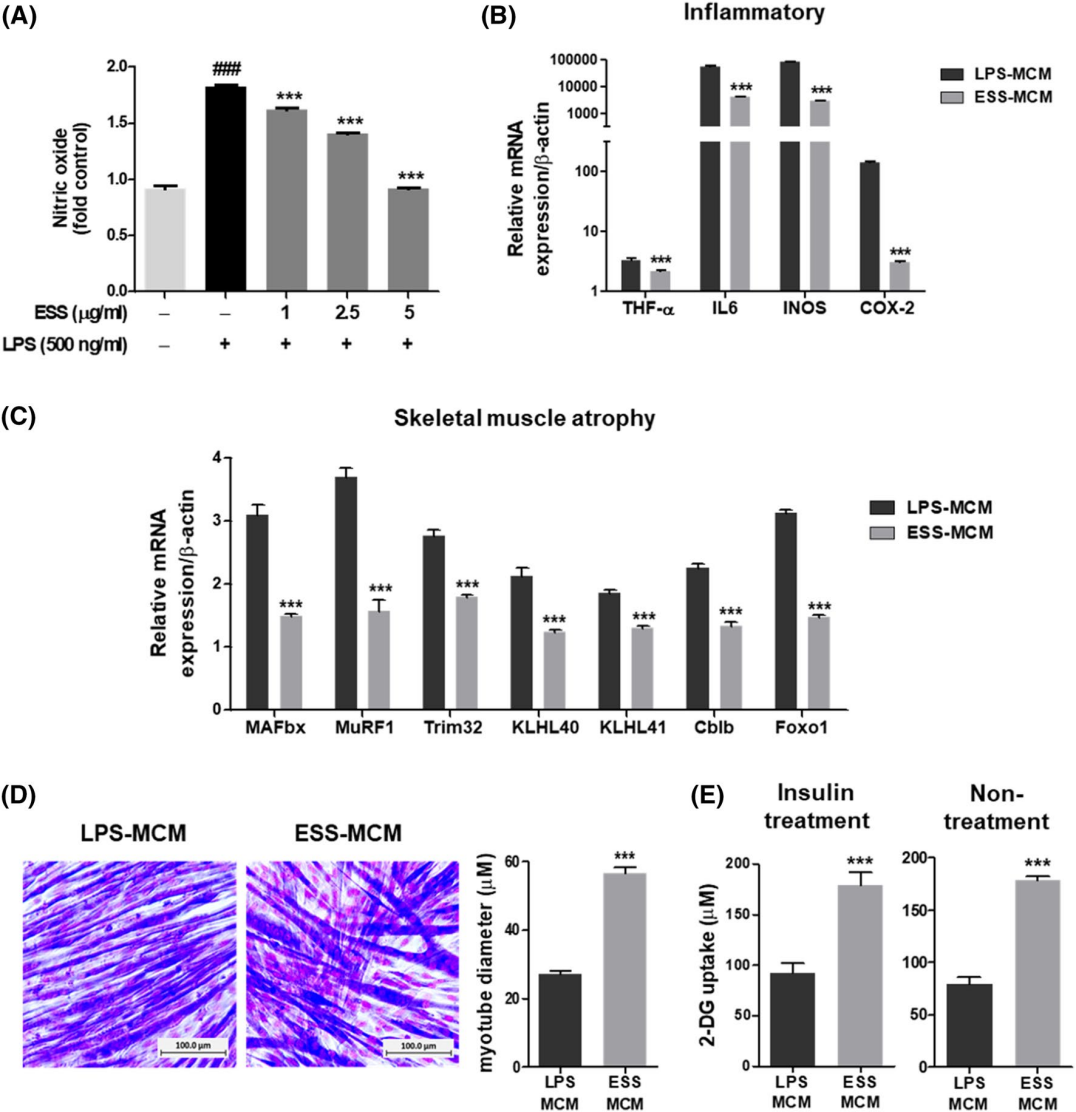
Anti-inflammatory Effects of ESS on RAW 264.7 Macrophages and the Influence of ESS-MCM on Muscle Function in C2C12 Myotubes. RAW 264.7 macrophages were pre-treated with ESS (1, 2.5, and 5 µg/ml) for 1 h and then exposed to LPS (500 ng/ml) for 24 h, assessed through the (**A**) NO assay, and expressed as a fold change relative to the control. C2C12 myotubes were treated with 20% MCM (LPS or ESS) for 24 h. Real-time PCR was employed to assess the mRNA expression levels of (**B**) inflammatory cytokines and mediators and (**C**) E3 ubiquitin ligase genes. (**D**) Representative images of myotubes captured at × 20 magnification illustrate the differences in muscle diameter between the LPS-MCM and ESS-MCM treatment groups. (**E**) Glucose uptake levels were measured in both insulin-treated and non-treated groups. The data are presented as mean ± SEM, with statistical analysis conducted for each gene. Figure A shows significance with ###*p* < 0.001 when compared to the untreated group and ***p < 0.001 when compared to the LPS-treated group. In Figures B to E, significance is indicated as ***p < 0.001 when compared to the LPS-MCM group.

In a prior study, ESS exhibited anti-inflammatory effects in RAW 264.7 macrophage cells^7^. In this investigation, we reassessed its anti-inflammatory efficacy at different concentrations (1, 2.5, and 5 µg/ml) using the NO assay for activity validation (Fig. 1A). The results demonstrated a significant reduction in NO levels induced by LPS (500 ng/mL) treatment in the presence of ESS. Subsequently, to assess the impact of macrophage-derived secreted factors on muscle function, we conducted experiments using differentiated C2C12 myotubes exposed to macrophage-conditioned medium treated with LPS (LPS-MCM) and ESS-treated macrophage-conditioned medium (ESS-MCM).

ESS-MCM treatment notably reduced the mRNA expression of inflammatory cytokines and mediators, such as tumor necrosis factor-α (TNF-α), interleukin (IL)-6, inducible nitric oxide synthase (iNOS), and cyclooxygenase-2 (COX-2) (Fig. 1B), thus mitigating the induction of inflammatory responses in muscle cells. Furthermore, our objective was to evaluate changes in muscle atrophy and inflammatory responses induced by these macrophage-derived secreted factors. Analysis of markers associated with the ubiquitin–proteasome pathway revealed significant downregulation of several E3 ubiquitin ligases, including MuRF1, MAFbx, Trim32, Cblb, KLHL40, and KLHL41, in response to ESS-MCM treatment, leading to the inactivation of the ubiquitin–proteasome pathway (Fig. 1C). Moreover, we observed a 63.3% decrease in cell diameter in myotubes treated with ESS-MCM compared to those treated with LPS-MCM, indicating a potential impact on muscle atrophy (Fig. 1D).

To investigate the influence of MCM on glucose uptake in C2C12 myotubes, we measured 2-deoxy-D-glucose (2-DG) uptake. Our results revealed a significant increase of 125.5% under insulin-stimulated conditions and 93.1% under non-insulin-stimulated conditions in the presence of ESS-MCM compared to LPS-MCM (Fig. 1E), indicating a substantial effect on glucose uptake and glycemic control. These findings emphasize the intricate role of macrophage-derived factors, particularly those from ESS-MCM, in modulating muscle physiology, inflammation, and glucose metabolism, highlighting potential avenues for further investigation.

### ESS-treated macrophages exhibit extensive alterations in gene expression patterns

**Figure 2:**
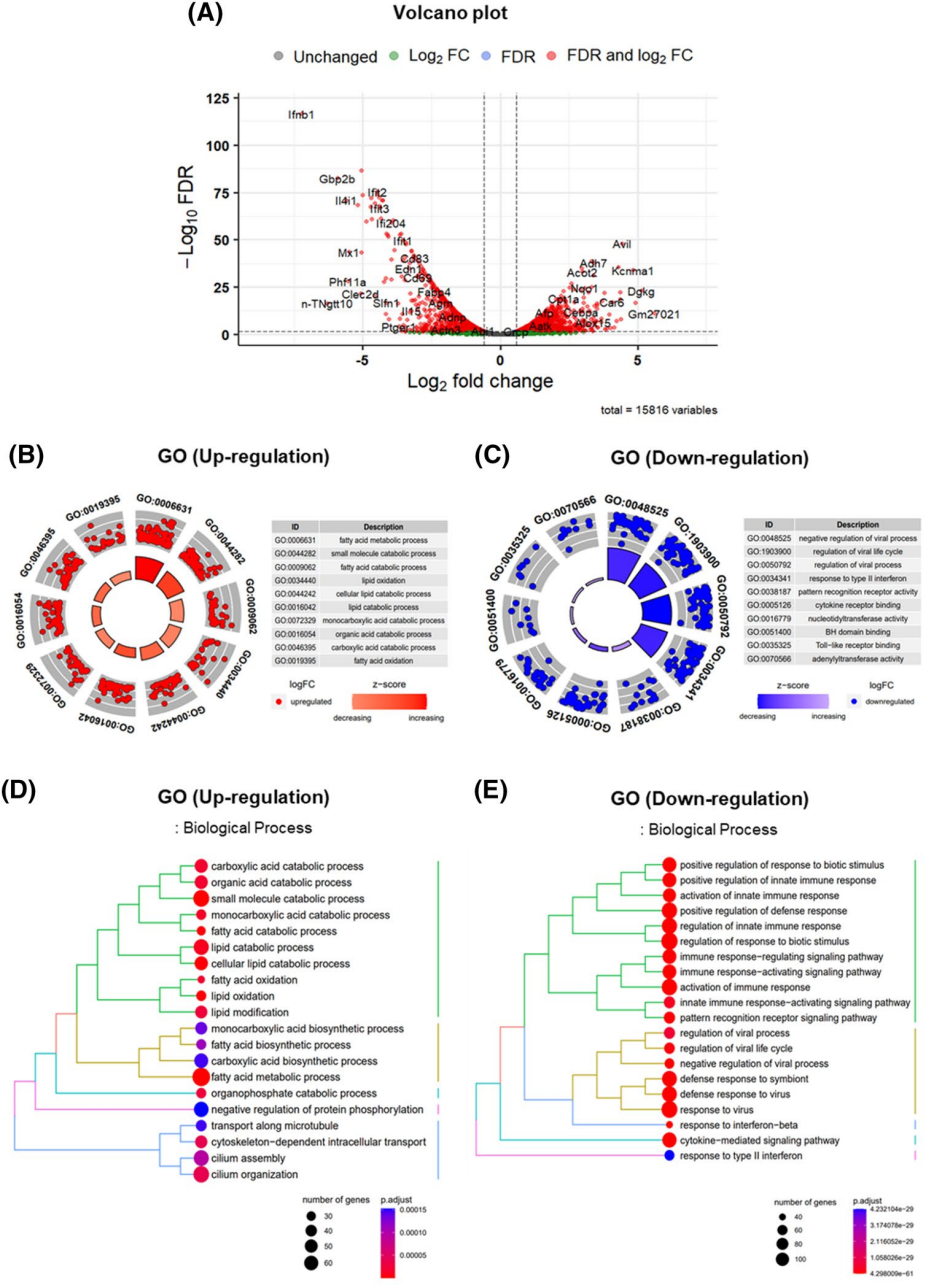
Analysis of Differentially Expressed Genes and Gene Ontologies (GOs) in Response to ESS Treatment in LPS-Stimulated Macrophages. (**A**) The volcano plot highlights 2019 up-regulated genes and 1333 down-regulated genes resulting from ESS treatment in LPS-stimulated macrophages. GO terms associated with the regulated genes were elucidated for both (**B**) upregulation and (**C**) downregulation. Furthermore, we conducted a focused examination of biological processes and identified them based on (**D**) upregulation and (**E**) downregulation. The presentation of the volcano plot and the GO analysis employed a threshold of |log_2_ FC|> = 0.58, and significance was determined at FDR < 0.05.

Given the significant effects of ESS-MCM on muscle mass and the associated gene expression changes affecting glucose homeostasis in C2C12 myotubes, we conducted an in-depth RNA-seq analysis to unveil the transcriptional alterations in macrophages exposed to both LPS and ESS. Our comprehensive investigation identified 3,352 genes with statistically significant differential expression (*P* < 0.05 and |log_2 _fold change (FC)|> = 0.58) in the ESS-treated group compared to the LPS group. Among these, 2,019 genes were up-regulated, with notably high expression levels observed for genes such as Ifnb1, Gbp2b, Il4i1, Ifit2, Ifit3, Ifi204, Ifit1, Mx1, Cd83, Edn1, Cd69, Phf11a, Clec2d, n-TNgtt10, Slfn1, Fabp4, Agrn, Il15, Adnp, Ptger1, Actn3, and Abi1. Conversely, 1,333 genes were downregulated, with prominent expression changes seen in genes like Avil, Adh7, Acot2, Kcnma1, Nqo1, Dgkg, Cpt1a, Car6, Afp, Cebpa, Gm27021, Aatk, Alox15, and Crcp (Fig. 2A).

To gain deeper insights into the modified functions of macrophages, we carried out Gene Ontology (GO) enrichment analysis on the differentially expressed genes (DEGs), covering biological processes (BP), molecular functions (MF), and cellular components (CC) (Supplementary Figure S1A,B). We focused on the top 10 enriched GO terms for upregulated and downregulated functions, respectively. The upregulated genes predominantly exhibited enhanced functions associated with BP terms, displaying significant enrichment in the following pathways: fatty acid metabolic process (GO:0006631), small molecule catabolic process (GO:0044282), fatty acid catabolic process (GO:0009062), lipid oxidation (GO:0034440), cellular lipid catabolic process (GO:0044242), lipid catabolic process (GO:0016042), monocarboxylic acid catabolic process (GO:0072329), organic acid catabolic process (GO:0016054), carboxylic acid catabolic process (GO:0046395), and fatty acid oxidation (GO:0019395) (Fig. 2B). Conversely, within the BP category, functions experiencing significant downregulation displayed noteworthy enrichments in negative regulation of viral process (GO:0048525), regulation of viral life cycle (GO:1903900), regulation of viral process (GO:0050792), response to type II interferon (GO:0034341), and pattern recognition receptor activity (GO:0038187). Additionally, within the MF category, enrichments were observed in cytokine receptor binding (GO:0005126), nucleotidyltransferase activity (GO:0016779), BH domain binding (GO:0051400), toll-like receptor binding (GO:0035325), and adenylyltransferase activity (GO:0070566) (Fig. 2C).

To explore the potential impact of macrophages on muscle and other tissues, we conducted a comprehensive analysis to identify enriched pathways in BP (Fig. 2D,E). In addition to pathways associated with immune responses, we observed a significant downregulation of the Cytokine-mediated signaling pathway. This observation suggests that macrophage-secreted factors may exert an influence on muscle tissue and other biological processes.

Moreover, during our pathway analysis utilizing the Kyoto Encyclopedia of Genes and Genomes (KEGG) database to identify enhanced pathways among the downregulated genes, we noted a substantial presence of pathways closely linked to immune systems and cytokine-cytokine receptor interactions. Specifically, the toll-like receptor signaling pathway, TNF signaling pathway, and NF-Kappa B signaling pathway emerged as prominent among the differentially expressed genes (Supplementary Figure S2A,B).

### Analysis of GO and KEGG network for secreted factors in ESS-treated macrophages

Considering the distinctive patterns of gene expression observed in macrophages when stimulated with ESS and LPS, particularly in the context of cytokines, secretory factors, and immune responses, we conducted an extensive network analysis to concurrently identify genes associated with both conditions. Our primary focus during this analysis was to pinpoint genes that are linked to the immune system responses in macrophages subjected to ESS treatment, encompassing the release of diverse factors, including cytokines and chemokines.

**Figure 3:**
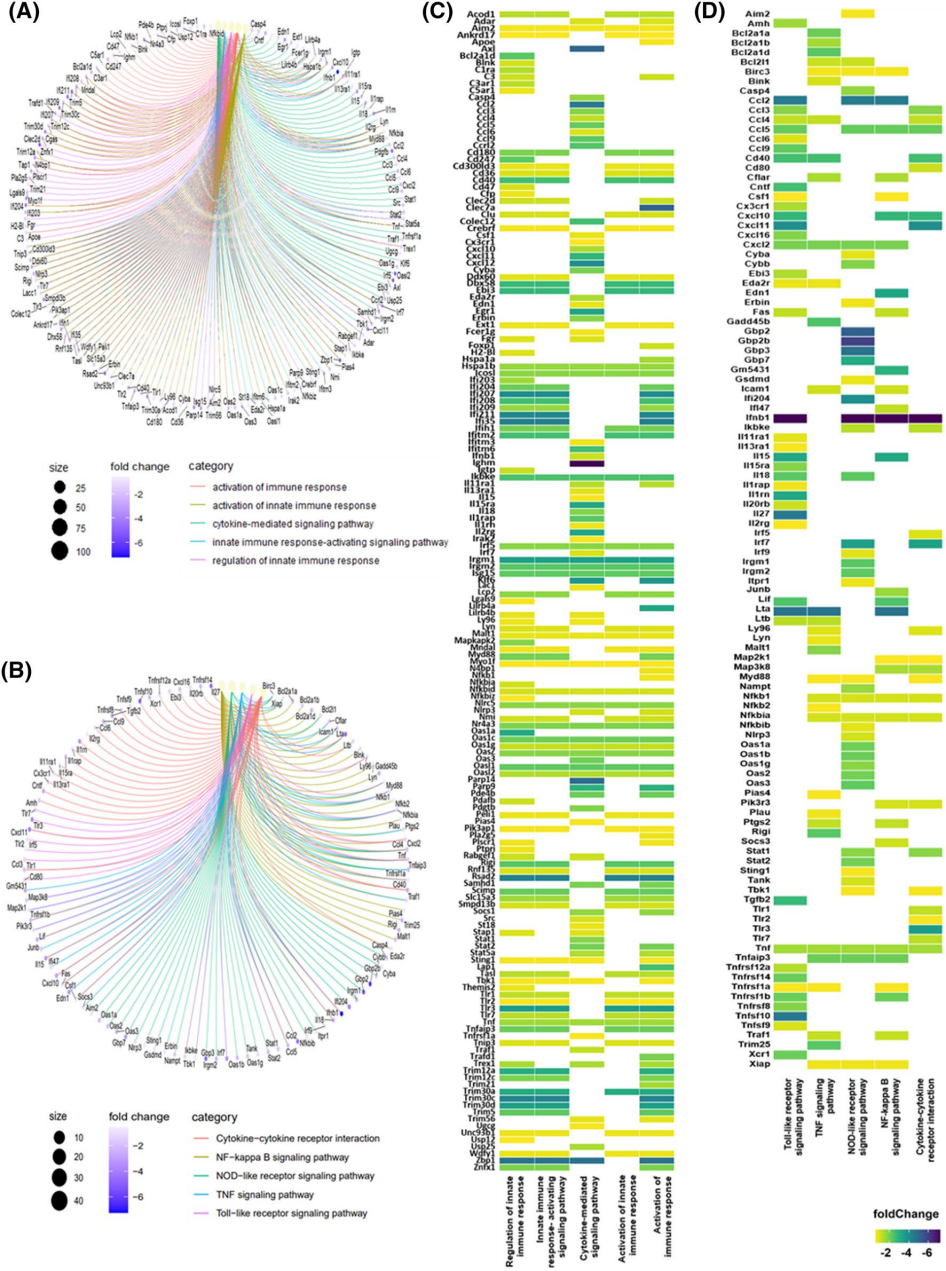
Network Analysis of Secreted Factors Related to GO Terms and KEGG Pathway Categories. Heatmaps were generated to visualize network analysis and gene expression patterns, with a specific emphasis on cytokine and chemokine terms identified in KEGG and GO terms. (**A**) Analysis of cytokine and immune system networks in GO terms and (**B**) KEGG pathways, along with illustrating gene expression patterns within (**C**) GO terms and (**D**) KEGG categories. Heatmaps were created using ClusterProfiler (version4.9.2, https://www.bioconductor.org/packages/devel/bioc/html/clusterProfiler.html). Data analysis was carried out using a threshold of |log_2_ FC|> = 0.58 and FDR < 0.05.

In the GO analysis, the "Activation of immune response" category, which involved the examination of 97 genes, revealed notable high-fold changes in genes like Ifi207, Ifi211, Ifi35, Rsad2, Trim30c, Zbp1, and Clec7a, among others. Similarly, in the "Activation of innate immune response" category, consisting of an analysis of 55 genes, genes such as Rsad2 displayed prominent fold changes. Moreover, the "Cytokine-mediated signaling pathway", analyzed with 85 genes, demonstrated noteworthy high-fold changes in genes like Axl, Ccl2, Cxcl12, Ighm, Parap14, and Zbp1. Likewise, both the "Innate Immune response-activating signaling pathway" (analyzing 72 genes) and "Regulation of innate immune response" (analyzing 100 genes) showcased genes such as Ifi207, Ifi211, Ifi35, Rsad2, Trim30c, and Zbp1, with high fold changes (Fig. 3A,C).

Turning to KEGG analysis, in the "Cytokine-cytokine receptor interaction" category, which involved 25 genes, notable fold changes were identified in genes like Cxcl11 and Ifnb1. Within the "NF-kappa B signaling pathway", comprising 30 genes, substantial high-fold changes were evident in genes like Ccl2, Ifnb1, Lta, and others. Additionally, the "NOD-like receptor signaling pathway", analyzed with 42 genes, showed substantial fold changes in genes like Gbp2, Gbp2b, Gbp7, and Ccl2. The "TNF signaling pathway", involving 32 genes, highlighted a high-fold change in Lta. Finally, in the "Toll-like receptor signaling pathway," encompassing 42 genes, notable fold changes were observed in genes like Tnfsf10, Lta, Il27, Ifnb1, Cxcl11, and Ccl2, among others (Fig. 3B,D). These findings collectively highlight the ability of ESS to induce significant changes in the immune system and secreted factors within LPS-activated macrophages.

### Verification of RNA-seq findings through real-time PCR analysis

**Figure 4:**
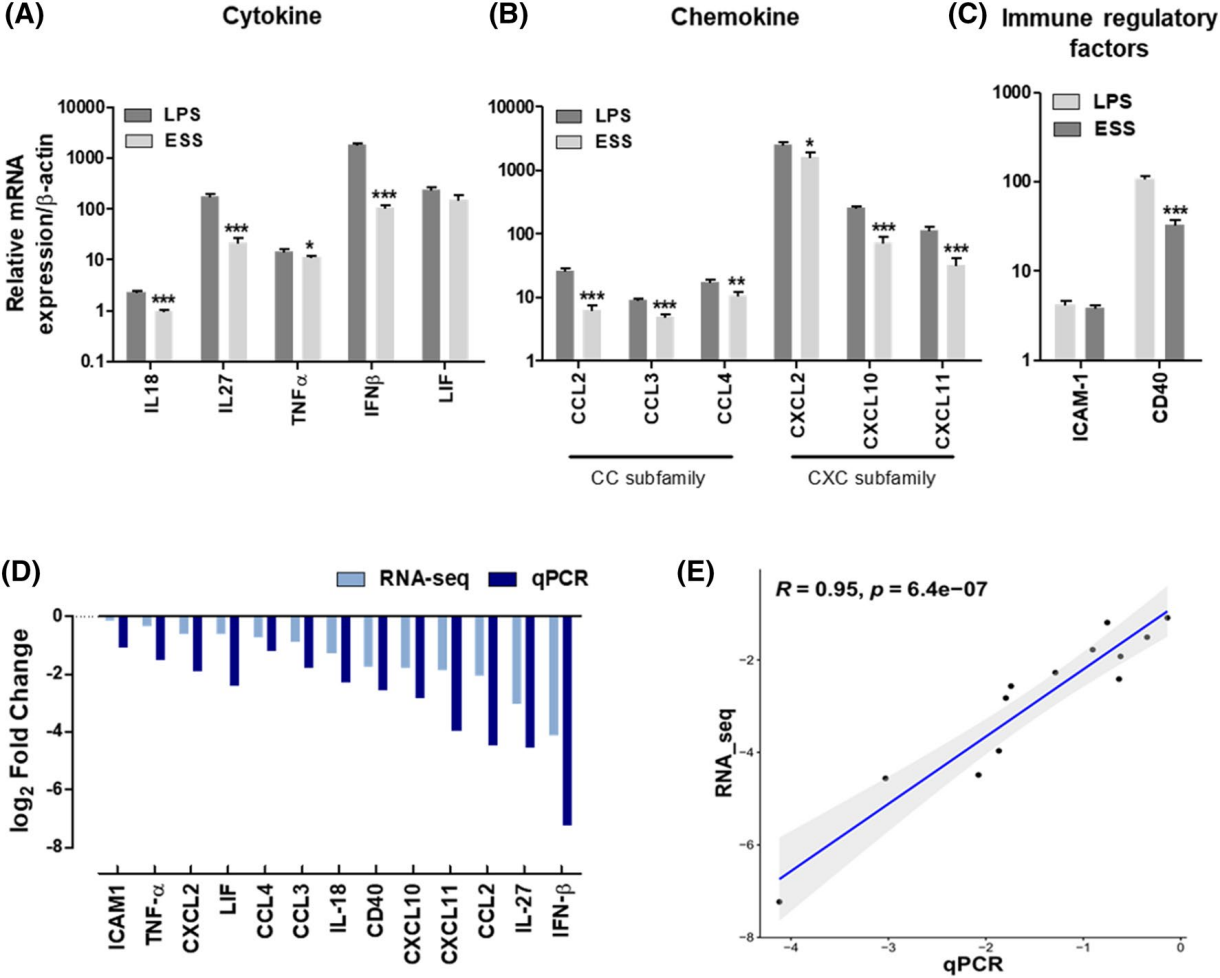
Validation of RNA-seq Results Using RT-qPCR. (**A**) Relative expression levels of cytokines, including IL-18, IL-27, TNF-α, IFN-β, and LIF, (**B**) chemokines, including CCL2, CCL3, CCL4, CXCL2, CXCL10, and CXCL11, and (**C**) immune regulators, including ICAM and CD40, determined by RT-qPCR. (**D**) Comparison of log_2_ FC between RNA-seq and RT-qPCR for selected genes. (**E**) Linear regression analysis of RNA-seq and RT-qPCR data shows a correlation coefficient (R) of 0.95. The data are presented as mean ± SEM. Statistical analysis was conducted separately for each gene, and significance levels were indicated as *p < 0.005, **p < 0.01, ***p < 0.001 compared to the LPS group.

To ascertain the robustness of the transcriptome sequencing data, an investigation utilizing real-time polymerase chain reaction (RT-qPCR) was carried out on a randomly chosen set of genes. These selected genes encompass a diverse spectrum of molecules, encompassing cytokines (Fig. 4A; IL-18, IL-27, TNF-α, IFN-β, and LIF), chemokines (Fig. 4B; CCL2, CCL3, CCL4, CXCL2, CXCL10, and CXCL11), and immune regulatory factors (Fig. 4C; ICAM-1 and CD40). The outcomes of the RT-qPCR analysis, as exemplified by the log_2 _FC values exhibited in Fig. 4D, consistently mirrored the expression patterns observed in RNA-seq data. Moreover, the correlation analysis yielded a correlation coefficient (R) of 0.95 (Fig. 4E), signifying a robust concordance between the two methodologies.

### Evaluation of secreted factors in MCM

**Figure 5:**
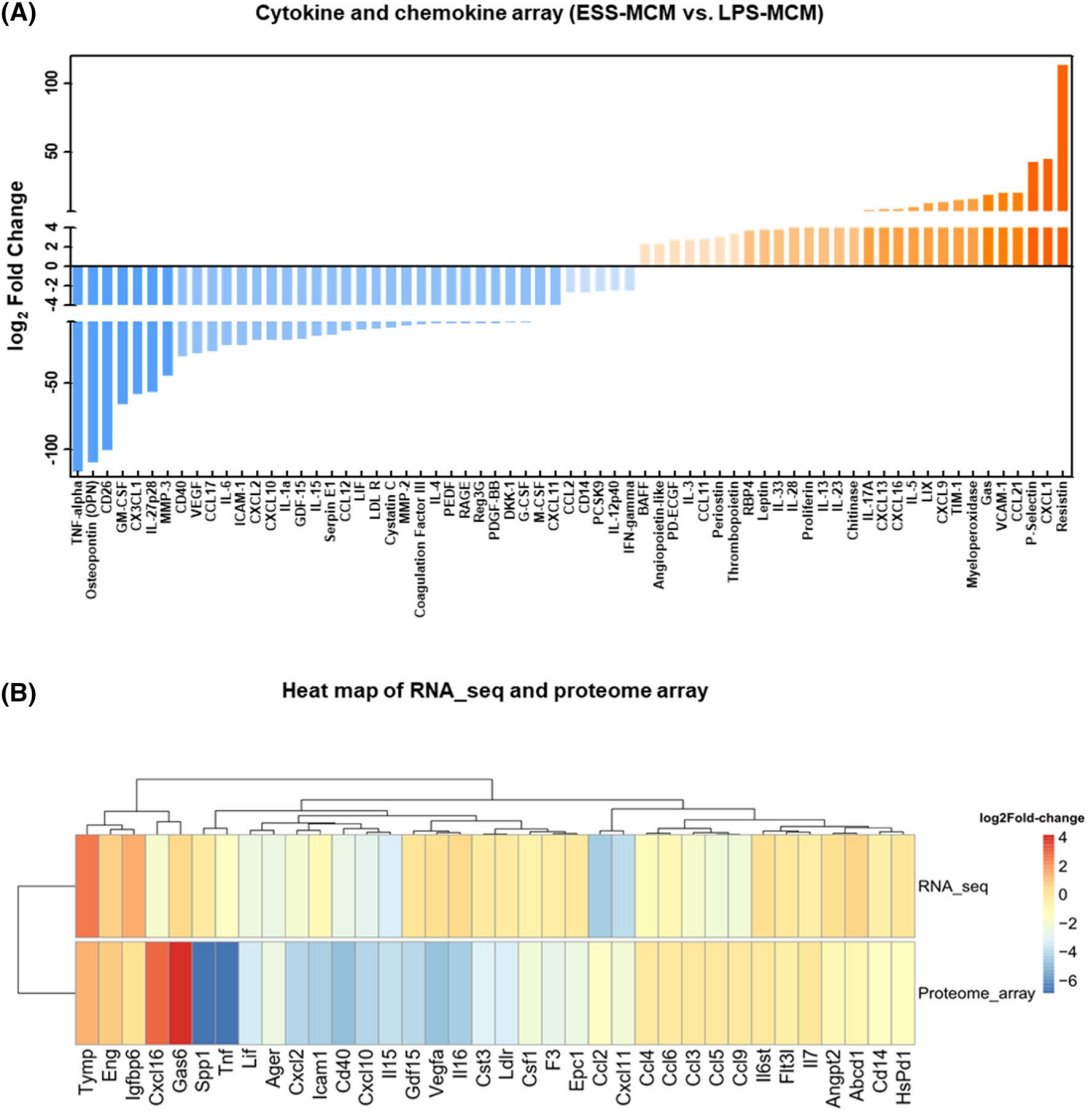
Analysis of Secreted Protein Arrays and Heatmap Comparison with RNA-seq. (**A**) Specific relative fold changes in secreted proteins were observed in ESS-MCM. (**B**) The levels of 36 secreted factors are depicted in a heatmap generated using Pheatmap (version 1.0.12, https://cran.r-project.org/web/packages/pheatmap/index.html).

To comprehensively evaluate the levels of secreted factors, including cytokines and chemokines, within MCM, we conducted a protein profiling analysis (Fig. 5A). Out of the 120 proteins examined, 29 factors exhibited upregulation, while 38 factors displayed downregulation (|log_2_ FC|≤ 2) in LPS-treated MCM. Notably, several factors associated with muscle atrophy, such as TNF-α, IL-6, IL-1, and GDF-15 (with log_2_ FC values of −116.8, −22, −17.6, and −16.8, respectively), showed significant reductions in ESS-MCM. Nevertheless, further exploration of the correlation in expression patterns of 36 secreted factors identified in both RNA-seq analysis and proteome array experiments, no close correlation was found (Fig. 5B).

### Anti-inflammatory effects of ESS main components: SQA, SHQA, and SCM in macrophage cells.

**Figure 6:**
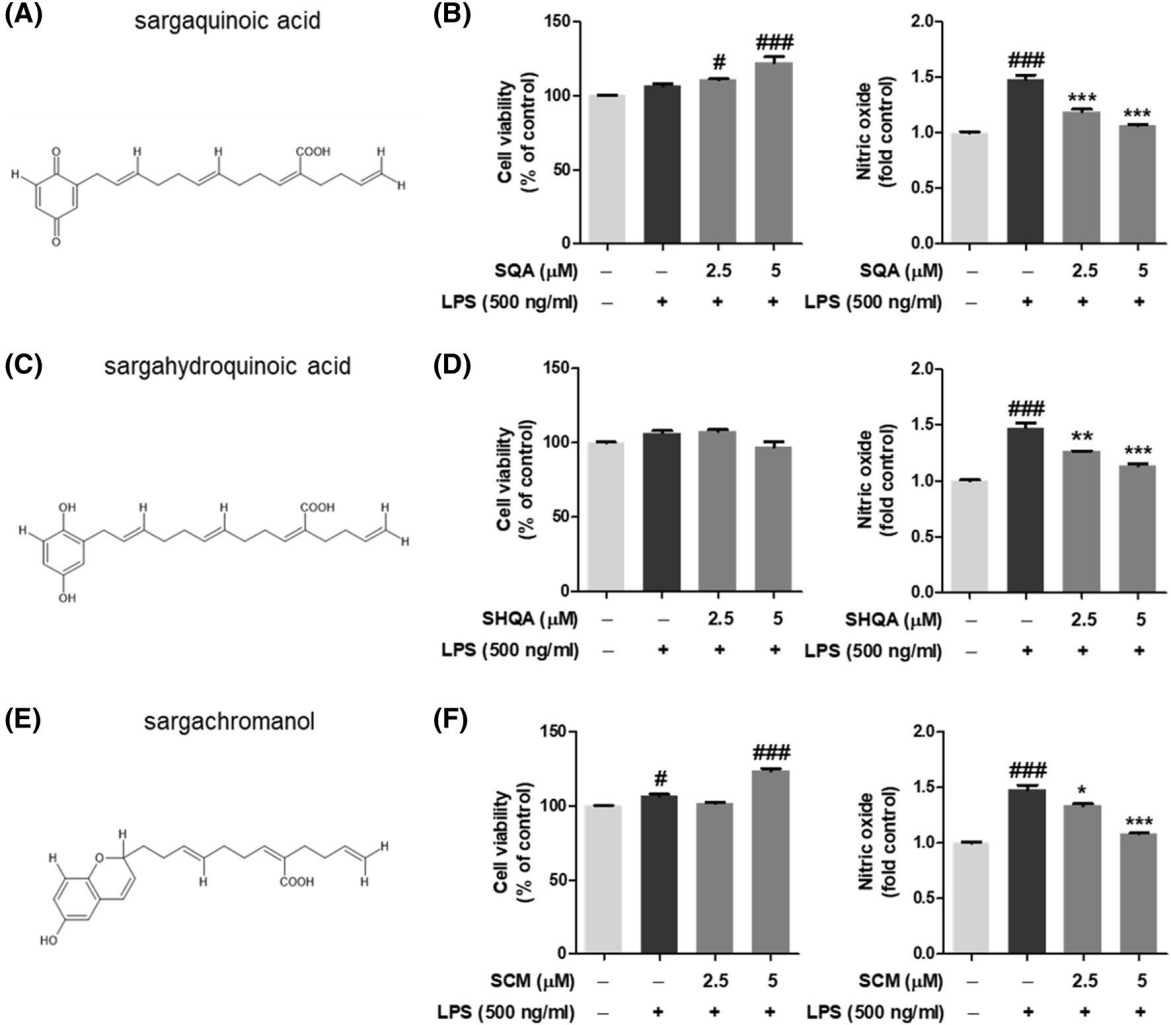
Assessment of Anti-inflammatory Effects of Main Components of ESS in RAW 264.7 Macrophages. Chemical structures of the major components of ESS were created (**A**) Sargaquinoic acid, (**C**) Sargahydroquinoic acid, and (**E**) Sargachromanol using Chemsketch software. RAW 264.7 cells were pre-treated with (**B**) SQA, (**D**) SHQA, and (**F**) SCM at concentrations of 2.5 and 5 μg/ml, respectively, for 1 h and then exposed to LPS for 24 h. Cytotoxicity was assessed using the MTS assay, and anti-inflammatory activity was determined by measuring NO production. The data is presented as mean ± SEM and statistical analysis was conducted for each gene. Significant differences are denoted as follows: #*p* < 0.005 and ###*p* < 0.001 when compared to the untreated group, and *p < 0.005, **p < 0.01, ***p < 0.001 when compared to the LPS-treated group.

Previous studies have identified the main components of ESS as sargachromanol (SCM), sargaquinoic acid (SQA), and sargahydroquinoic acid (SHQA)^7,8^. To determine which components significantly contribute to the regulation of macrophage transcripts and secretory factors, we assessed cell viability in RAW 264.7 macrophages stimulated with LPS and simultaneously evaluated their anti-inflammatory effects using a NO assay. First, SQA (Fig. 6A) increased cell viability and markedly reduced NO levels, demonstrating anti-inflammatory properties (Fig. 6B). SHQA (Fig. 6C) showed no toxicity in terms of cell viability up to a concentration of 5 µM and displayed anti-inflammatory effects by significantly reducing NO production (Fig. 6D). Lastly, SCM (Fig. 6E) increased cell viability at a concentration of 5 µM and significantly reduced NO production, indicating anti-inflammatory effects (Fig. 6F). These findings underscore that the sargassum-derived compounds SQA, SHQA, and SCM, extracted from ESS, collectively demonstrate anti-inflammatory characteristics akin to those of ESS itself when administered to macrophages.

## Discussion

The research presented in this study represents a significant advancement in our ongoing exploration of the intricate interplay between inflammation, cellular metabolism, and potential therapeutic strategies. In recent years, the scientific community has increasingly recognized the pivotal role that macrophages play in orchestrating immune responses and their substantial impact on a wide range of physiological processes. This investigation delves deeply into the dynamic interaction between macrophages and ESS, a substance that has garnered substantial attention for its anti-inflammatory properties and its ability to regulate metabolic processes. Within the framework of this comprehensive research, the study yields profound insights.

These findings offer valuable insights into the intricate connections among cytokines produced by macrophages and their effects on muscle physiology, inflammation, and glucose metabolism. The primary aim of this study was to assess the impact of two distinct macrophage secretion media on muscle function: one group exposed to LPS-MCM and another exposed to ESS-MCM.

One of the most significant discoveries in this study is the association between ESS and muscle health. Following ESS-MCM treatment, a substantial downregulation of E3 ubiquitin ligases, including MuRF1, MAFbx, Trim32, Cblb, KLHL40, and KLHL41, was observed. These downregulations suggest the potential inhibition of the ubiquitin–proteasome pathway, a major pathway implicated in muscle atrophy. Moreover, the reduced expression of inflammatory cytokines and mediators in ESS-MCM-treated cells indicates the suppression of inflammatory responses in muscle cells, suggesting that ESS-MCM may confer protective effects against muscle atrophy and inflammation. Another significant finding is the notable reduction in the cell diameter of myotubes treated with ESS-MCM compared to those treated with LPS-MCM, suggesting potential implications for muscle atrophy.

Moreover, glucose uptake was significantly elevated in the presence of ESS-MCM under both insulin-stimulated and non-insulin-stimulated conditions, underscoring its substantial impact on glucose metabolism. However, consistent similarities in glucose uptake between insulin-stimulated and unstimulated conditions were observed across repeated experiments, indicating potential deviations from established norms in the insulin-related glucose uptake response. The regulation of glucose uptake in skeletal muscle and adipocytes primarily involves insulin-mediated translocation of the glucose transporter GLUT4 from intracellular vesicles to the cell surface, a critical process facilitating glucose uptake. GLUT4 expression serves as a cornerstone of the insulin-responsive vesicle compartment and plays a pivotal role in insulin sensitivity^16^. A study examining the effects of dexamethasone treatment on GLUT4 expression and insulin-stimulated glucose transport mediated through the glucocorticoid receptor lends support to the observed similarities in glucose uptake between insulin-stimulated and unstimulated conditions^17^. Despite the significant increase in GLUT4 expression induced by dexamethasone, there was no corresponding enhancement in insulin-stimulated glucose transport in C2C12 cells. Additionally, intact signaling from the insulin receptor to its downstream target Akt2 was observed in dexamethasone-treated cells, and the expression of SNARE proteins involved in vesicle trafficking remained normal^17^. This suggests insulin-dependent trafficking of insulin-responsive markers, including insulin-responsive aminopeptidase and transferrin receptor. These findings support the hypothesis that increased GLUT4 expression alone is insufficient to establish an insulin-sensitive vesicular compartment. While increases in GLUT4 expression can occur in response to specific stimuli, such as dexamethasone, the ability of cells to respond to insulin and effectively transport GLUT4 to the cell surface for glucose uptake is a multifaceted process dependent on the integrity of the insulin-responsive endoplasmic reticulum trafficking machinery. Therefore, the observed similarities in glucose uptake between insulin-stimulated and unstimulated conditions in C2C12 cells in our study may be attributed to factors such as the complex regulation of the vesicle transport machinery, extending beyond GLUT4 expression. This interpretation underscores the importance of considering not only GLUT4 expression but also the functional integrity of the insulin-responsive vesicle compartment when evaluating the glucose uptake capacity of skeletal muscle cells.

The RNA-seq analysis of macrophages exposed to LPS and ESS unveiled extensive alterations in gene expression patterns. A comprehensive understanding of these gene expression changes was elucidated through GO enrichment analysis, shedding light on the functional significance of these gene expression modifications. Upregulated genes were predominantly associated with metabolic processes, encompassing fatty acid metabolism and oxidation pathways, while downregulated genes were linked to immune responses, virus regulation, and cytokine receptor binding. Our primary focus centers on the investigation of the down-regulation of the GO category of cytokine receptor binding. Nevertheless, it is of paramount importance that we delve into the intricate role played by lipid metabolism in macrophages about their up-regulation. It is noteworthy that the disparities between M1 and M2 macrophages extend to their distinctive demands in the realm of fatty acid metabolism, with M1 macrophages reliant on fatty acid synthesis (FAS), while M2 macrophages are closely linked to anti-inflammatory functions^18^. Recent research endeavors have cast a spotlight on the pivotal role of fatty acid oxidation (FAO) in shaping the phenotype of macrophages and its ability to quell inflammatory signals^19^. Notably, FAO has been intriguingly associated with the lifespan of cells, potentially exerting an influence on the endurance of tissue-resident macrophages^20^. Furthermore, the prospect of augmenting FAO, particularly in conditions such as atherosclerosis, holds promise for mitigating lipid accumulation and curbing the production of pro-inflammatory cytokines, thus offering promising avenues for therapeutic intervention^21^. These revelations underscore the critical significance of disrupted lipid metabolism in macrophages as a contributing factor to the pathogenesis of various diseases. This raises the possibility that reinforcing FAO through interventions like enhanced substrate supply, such as ESS in fatty acid metabolism, may have relevance for diseases with similar metabolic foundations. Consequently, Further research in this direction is imperative for a comprehensive understanding of the potential therapeutic applications of bolstering FAO in macrophages.

KEGG analysis highlighted the crucial roles of toll-like receptor signaling, TNF signaling, and NF-Kappa B signaling pathways in the response to ESS treatment, underscoring their pivotal role in mediating responses to ESS. Pathway analysis also indicated downregulation in cytokine-mediated signaling pathways, suggesting that factors secreted by macrophages could impact not only muscle tissues but also immune responses. Separate investigations have demonstrated that ESS inhibits the nuclear translocation and activation of the NF-κB/p65 subunit, indicating that ESS regulates the inhibition of inflammatory protein expression in cells stimulated with LPS^7^. These outcomes align with the trends observed in RNA-seq data, accentuating the regulatory role of ESS in suppressing inflammatory protein expression in cells stimulated with LPS.

In our comprehensive analysis, the scrutiny of protein profiles within MCM has unveiled significant alterations in the levels of secreted factors, with a particular emphasis on cytokines and chemokines. Remarkably, several factors associated with muscle atrophy exhibited substantial reductions in ESS-MCM, underscoring their potential role in mitigating ESS-induced muscle atrophy. Drawing upon data derived from protein array analysis, we have observed distinct regulatory patterns among various cytokines when comparing ESS-MCM to LPS-MCM. In this regard, there exists a substantial body of research that has shed light on the impact of TNF-α^22,23^, IL-6^24,25^, IL-1^26^, and GDF-15^27,28^—all markedly reduced in ESS-MCM—on muscle atrophy and muscle insulin resistance. Elevated TNF-α levels are well-documented in various diseases and are known to promote muscle breakdown^22^. TNF-α induces muscle wasting through the activation of the ubiquitin–proteasome pathway responsible for muscle protein degradation. Additionally, it upregulates atrogin1/MAFbx, a crucial ubiquitin ligase for muscle breakdown. The p38 MAPK signaling pathway plays a central role in mediating TNF-α-induced muscle catabolism, potentially interacting with Foxo transcription factors and other relevant pathways^23^. IL-6 has been definitively established as detrimental to muscle tissue. Prolonged elevation of IL-6 can lead to both muscle atrophy and insulin resistance. It activates the ubiquitin–proteasome system, initiating muscle protein degradation and interfering with muscle cell differentiation and fusion. In terms of insulin resistance, IL-6 disrupts insulin signaling pathways through the phosphorylation of insulin receptor substrate proteins and activation of serine kinases like JNK^24,25^. IL-1 is an inflammatory cytokine associated with muscle catabolism, regulating the expression of E3 proteins in muscles, including MAFbx and MuRF1. Their study is findings demonstrate that both IL-1α and IL-1β directly impact muscles, leading to increased expression of atrogin1/MAFbx and MuRF1 mRNA, concurrent with a reduction in specific muscle proteins and muscle fiber size. These responses are closely linked to the upregulation of E3 ligase expression and the stimulation of muscle catabolism. These results underscore IL-1's capacity to modulate muscle catabolism, whether through direct or indirect mechanisms, within the confines of muscle tissue^26^.GDF-15, a stress-responsive cytokine, may contribute to muscle atrophy and insulin resistance by inducing muscle protein breakdown, primarily through the Bcl-2/caspase-3 pathway. While its direct impact on muscle insulin resistance is not fully understood, elevated GDF-15 levels are associated with insulin resistance in various tissues, indirectly affecting muscle insulin sensitivity^27,28^. These findings underscore the differential regulation of secreted factors encompassing cytokines and chemokines, which play a central role in muscle atrophy and glucose metabolism impairments. The downregulation of TNF-α, IL-6, IL-1, and GDF-15 in ESS-MCM, particularly in their well-established roles in promoting muscle atrophy and insulin resistance, is of paramount significance. This study highlights the intricate interplay between the macrophage transcriptome and secreted factors from macrophages, which can have either detrimental or beneficial effects on skeletal muscle function. Notably, ESS treatment shows promise in reducing macrophage-derived factors, indicating a significant potential role in preserving skeletal muscle function.

## Method and material

### Materials

LPS (Escherichia coli O55:B5), dimethyl sulfoxide (DMSO), Phosphoric acid, Sulfanilamide, N-(1-Naphthyl) ethylenediamine dihydrochloride (NED), and Giemsa solution were obtained from Sigma-Aldrich. The 4% Paraformaldehyde Phosphate Buffer Solution was purchased from Fujifilm WAKO (Richmond, VA, USA). Additionally, the RiboEx™ reagent was acquired from GeneAll (Seoul, Korea). The supplies including Dulbecco’s Modified Eagle's Medium (DMEM), fetal bovine serum (FBS), penicillin, and streptomycin (P/S), as well as Phosphate-Buffered Saline (PBS), were purchased from Welgene (Daegu, Korea). The chemical structures of all these molecules were drawn by using ACD/ChemSketch software (version 2023, https://www.acdlabs.com/resources/free-chemistry-software-apps/chemsketch-freeware/). *S. serratifolium* ethanol extract (ESS) samples were prepared based on a previous publication and dissolved in DMSO at various concentrations^7^.

### Cell culture and sample treatment

RAW 264.7 macrophage cells (sourced from the Korea Cell Line Bank) were seeded in a 12-well plate at a density of 4 × 10^5 cells per well. These cells were then incubated overnight in DMEM supplemented with 10% FBS and 1% P/S. To prepare macrophage-conditioned 5media (MCM), the cells underwent pretreatment with ESS (3 μg/ml, either treated or untreated) for 2 h. Following this pretreatment, the cells were stimulated with LPS (500 ng/ml) for 3 h. ESS was dissolved in DMSO for cell processing, ensuring a final DMSO concentration below 0.1% in each treatment. After the designated treatment period, the cells were subjected to two washes with DPBS, and serum-free medium was added for an additional 24 h to facilitate the collection of MCM (Supplementary Figure S3). The collected media were then subjected to centrifugation at 1,000 rpm for 3 min to eliminate cellular debris, and the resulting supernatant was preserved at −80 °C for subsequent experiments.

C2C12 skeletal muscle cells (obtained from ATCC, Rockville, MD, USA) were seeded in 6-well plates at a density of 2 × 10^5 cells per well and were cultured in DMEM supplemented with 10% FBS and 1% P/S, following established protocols. To initiate myotube differentiation, the serum concentration was reduced to 2% FBS, and the culture medium was refreshed every 2 days. On the 5th day of the differentiation process, the cells were exposed to MCM (20%) for 24 h to facilitate the experimental procedures. Throughout all stages, incubation was sustained within a humidified environment at 37 °C with 5% CO2.

### Cell viability assay

Cell viability was assessed using the MTS assay (CellTiter96® AQueous One Solution Cell Proliferation Assay Kit, Promega, Madison, WI, USA). To summarize the procedure, RAW 264.7 cells (4 × 10^4^ cells/well) were initially seeded in a 96-well plate. Following a 24-h incubation, the cells were cultured for an additional 24 h with ESS or individual ESS compounds (SCM, SQA, SHQA) that were diluted in serum-free medium at various concentrations. After the incubation period, 5 μL of MTS reagent was added to each well, and the color was allowed to develop for 1 h at 37 °C. The absorbance was subsequently measured at 490 nm using a microplate reader (AMR-100, Allsheng, Hangzhou, China).

### Nitric oxide assay

RAW 264.7 macrophage cells were initially plated in 96-well plates at a density of 4 × 10^4^ cells per well. Following this, the cells were cultured overnight in DMEM (supplemented with 10% FBS and 1% P/S) under humidified conditions at 37 °C with 5% CO2. After a 24-h incubation period, when the cells reached 80% confluence, they underwent pretreatment with ESS, which was diluted in serum-free media. After one hour of pretreatment, the cells were exposed to LPS (500 ng/mL) for an additional 24 h. Subsequently, the supernatant was mixed in a 1:1 ratio with Griess reagent (comprising 8.5% Phosphoric acid, 1% Sulfanilamide, and 0.1% naphthyl ethylenediamine dihydrochloride dissolved in deionized distilled water) for 30 min, and the absorbance was measured at 540 nm using a microplate reader.

### Myotube diameter measurement

The diameters of C2C12 differentiated myotubes were determined using Giemsa staining. After the cells were fixed with a 4% Paraformaldehyde Phosphate Buffer Solution for 10 min, they were stained with Giemsa solution for 45 min. Following thorough washing with distilled water, the cells were air-dried, and a minimum of 6 random images were captured from each well. Image Processing and Analysis in Java (ImageJ) software (version 1.53, https://imagej.net/ij/) was employed to measure the diameter of 50 to 100 myotubes in each well.

### 2-Deoxy glucose uptake

For the measurement of glucose uptake, the Glucose Uptake Assay Kit (ab136955, Abcam, Cambridge, UK) was employed, following the provided protocol. Upon differentiation, C2C12 cells were subjected to a 24-h treatment with MCMs, followed by a 40-min starvation period using Krebs–Ringer-Phosphate-Hepes (KRPH) buffer. This buffer contained 20 mM HEPES, 5 mM KH2PO4, 1 mM MgSO4, 1 mM CaCl2, 136 mM NaCl, and 4.7 mM KCl, adjusted to pH 7.4. Post the starvation phase, the cells were either stimulated with 100 nM insulin for 20 min or left unstimulated. Subsequently, they were incubated with 10 μl of 2-deoxy-glucose (2-DG) for 20 min. 2-DG-6-phosphate (2-DG6P) oxidation reaction to NADPH was quantified at 412 nm using a microplate at 37 °C.

### Real time-PCR

RNA extraction from the cells was carried out using the RiboEx™ reagent (GeneAll, Seoul, Korea) following the manufacturer's protocol. Subsequently, the extracted RNA was converted into cDNA using the SmartGene compact cDNA Synthesis kit (SMART GENE, Daejeon, Korea). For real-time PCR, the TOPreal™ SYBR Green qPCR PreMIX (Enzynomics, Daejeon, Korea) was utilized, and the amplification was performed on a QuantStudio™ 1 Real-Time PCR system (Applied Biosystems, Foster City, CA, USA). To determine the expression levels of the target genes, normalization was done with the housekeeping gene beta-actin, and the fold change was calculated using the 2-ΔΔCT method^29^.Primer sequences are provided in Supplementary Table 1.

### RNA purification and sequencing

RNA purification and sequencing were performed using the same methods as previous studies^30^. Briefly, samples with an RNA Integrity Number (RIN) value of 7 or higher were used to construct libraries following the manufacturer's instructions with the Illumina TruSeqStranded Total RNA Library Prep Gold Kit (Illumina, San Diego, CA, USA, #20,020,599). From the purified samples, cDNA libraries were synthesized and quantified following the qPCR Quantification Protocol Guide (KAPA BIOSYSTEMS, #KK4854) by connecting adapters to the cDNA through adapter ligation. Sequencing was performed on the Illumina NovaSeq6000 (Illumina, Inc., San Diego, CA, USA). After removing low-quality adapter sequences, mapping to the reference genome was carried out using HISAT (version 2.1.0)^31,32^. Subsequently, read counting was performed using StringTie (version 2.1.3b)^31,33^.

### Analysis of differentially expressed genes

To identify differentially expressed genes, TMM normalization was conducted using the edgeR package (version 3.40). The TMM normalization utilized the No Replicate method as described in the "edgeR: Differential Analysis of Sequence Read Count Data User's Guide," with a square root dispersion of 0.1. Differentially expressed genes were filtered based on |log_2_ fold change (FC)|> = 0.58 and FDR < 0.05, and then used for GO and KEGG enrichment analyses, with the latter utilizing the Kanehisa laboratory's database^34–36^.

### Cytokine and chemokine profile analysis

Cytokine and chemokine levels were examined utilizing the Proteome Profiler Mouse XL Cytokine Array Kit (R&D, Minneapolis, MN, USA), and the experimental procedure followed the manufacturer's instructions. Initially, the membranes were subjected to a 1 h incubation at room temperature with a blocking buffer. Next, the membrane was exposed to the biotinylated Detection Antibody Cocktail and MCMs overnight, maintaining a temperature range of 2–8 °C. After thorough washing, a 30 min incubation with Streptavidin-HRP was conducted at room temperature. Finally, the membranes were treated with Chemi Reagent Mix for 1 min and exposed to X-ray film for 10 min. To quantify the results, the obtained images were analyzed using the specialized Quick Spots Tool software (version 25.6.0.0, https://idealeyes.com/products/QuickSpots/). The levels of cytokines and chemokines were determined as signal intensities normalized to the reference spots present on the same membrane (Supplementary Figure S4A,B).

### Statistical analysis

The mean ± standard error of the mean (SEM) was used to express the experimental results, which were derived from data collected through at least three independent trials. To analyze the significance between groups, GraphPad Prism software (version 5.0, https://www.graphpad.com/) was employed. One-way analysis of variance (ANOVA) in combination with Tukey’s multiple comparison test was used for overall comparisons among groups. Additionally, to compare specific pairs of groups, a two-sample t-test was performed to determine their significance.

### Supplementary Information

Supplementary Information.

## Data Availability

The sequencing data have been deposited in the NCBI Sequence Read Archive (SRA) database under the accession code PRJNA1061066 (Accession Numbers. SRR27420173 and SRR27420174). SRA records will be accessible with the following link after the indicated release date: https://www.ncbi.nlm.nih.gov/sra/PRJNA1061066.
