# Supplementary material for: Modulation of macrophage transcript and secretion profiles by Sargassum Serratifolium extract is associated with the suppression of muscle atrophy
Source: Sci Rep. 2024 Jun 10;14:13282. doi: 10.1038/s41598-024-63146-0 (PMC11165015; doi:10.1038/s41598-024-63146-0)
Supplement: Supplementary file 1 — Supplementary Information. [file 41598_2024_63146_MOESM1_ESM.docx]

**Supplementary Figure 1.**


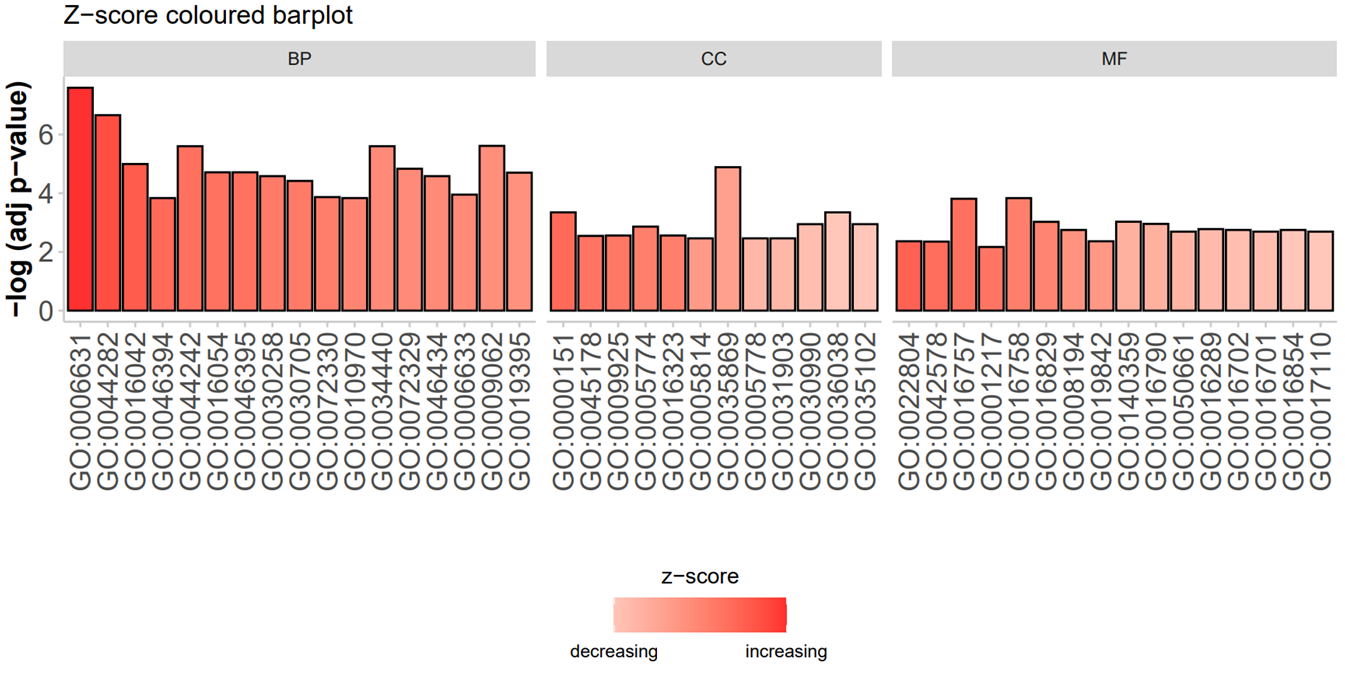

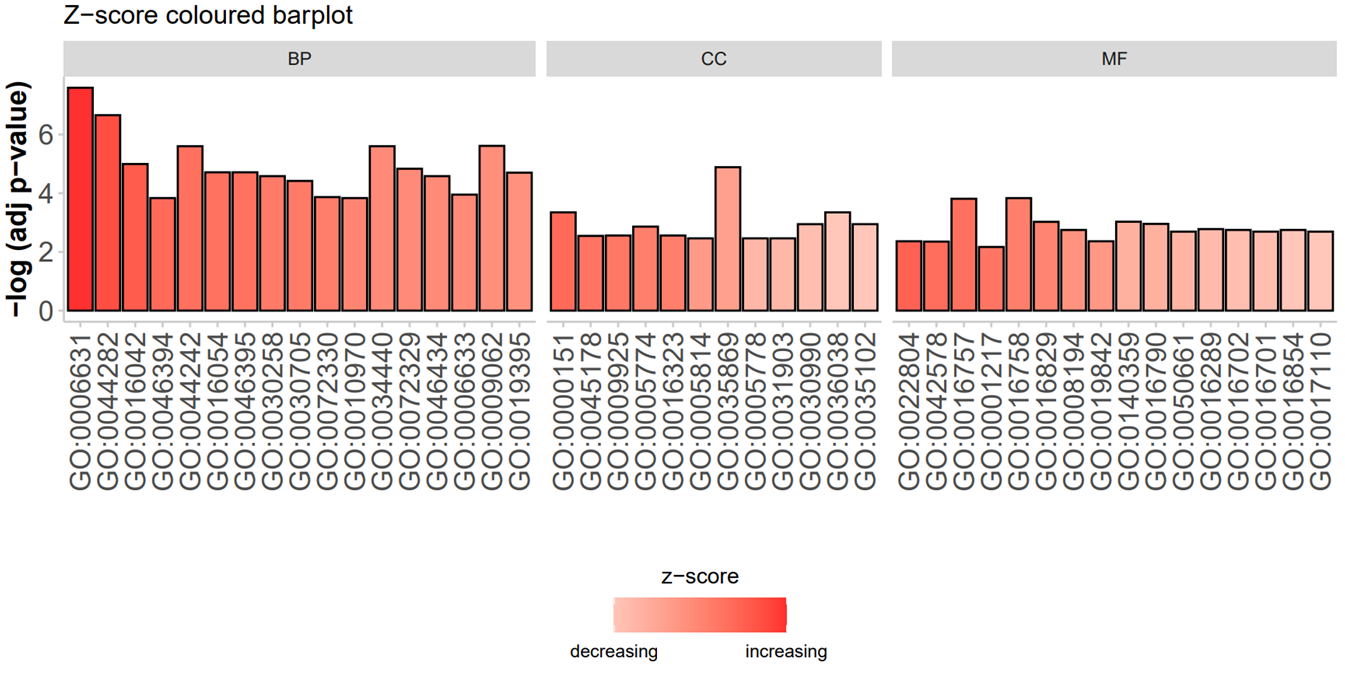

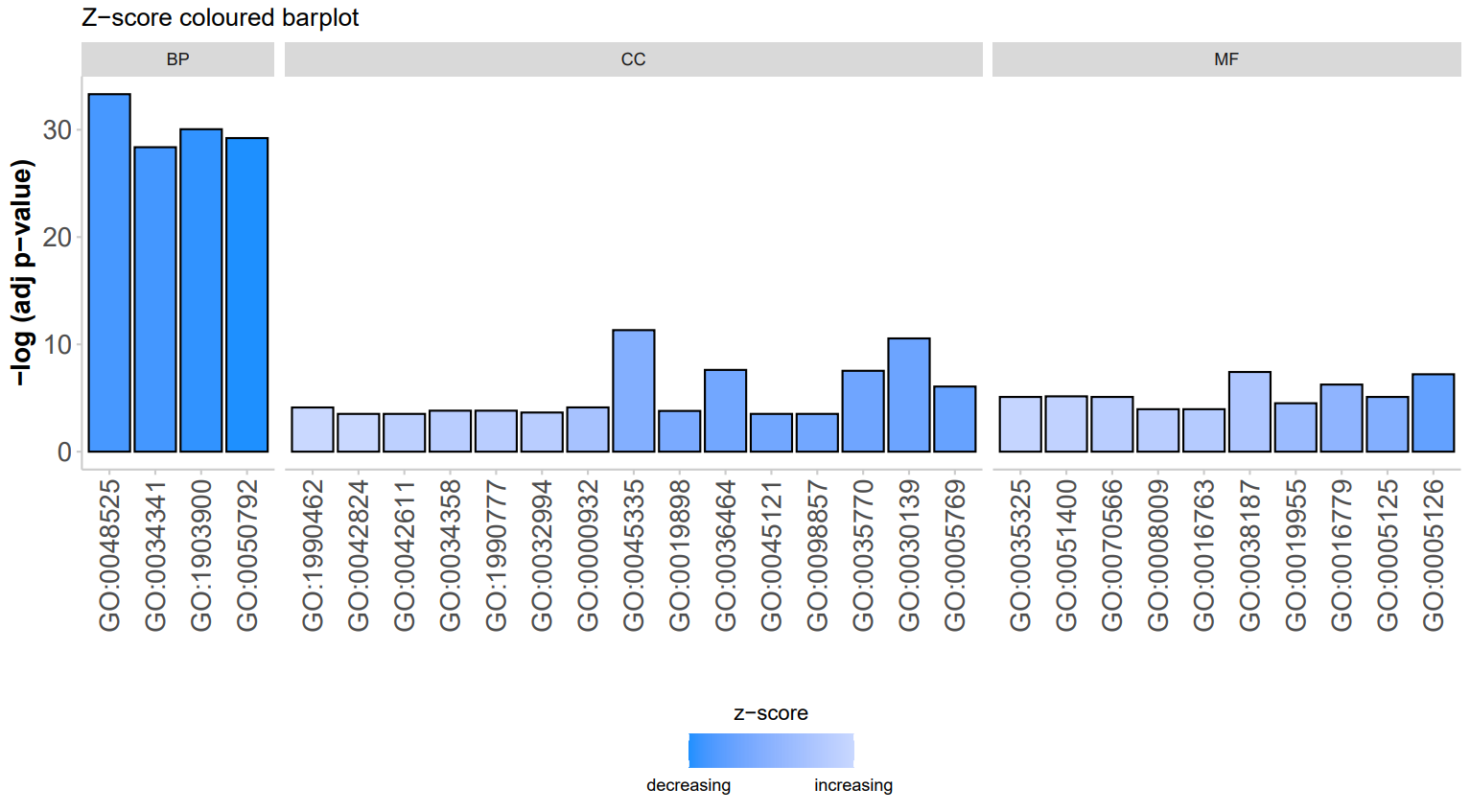

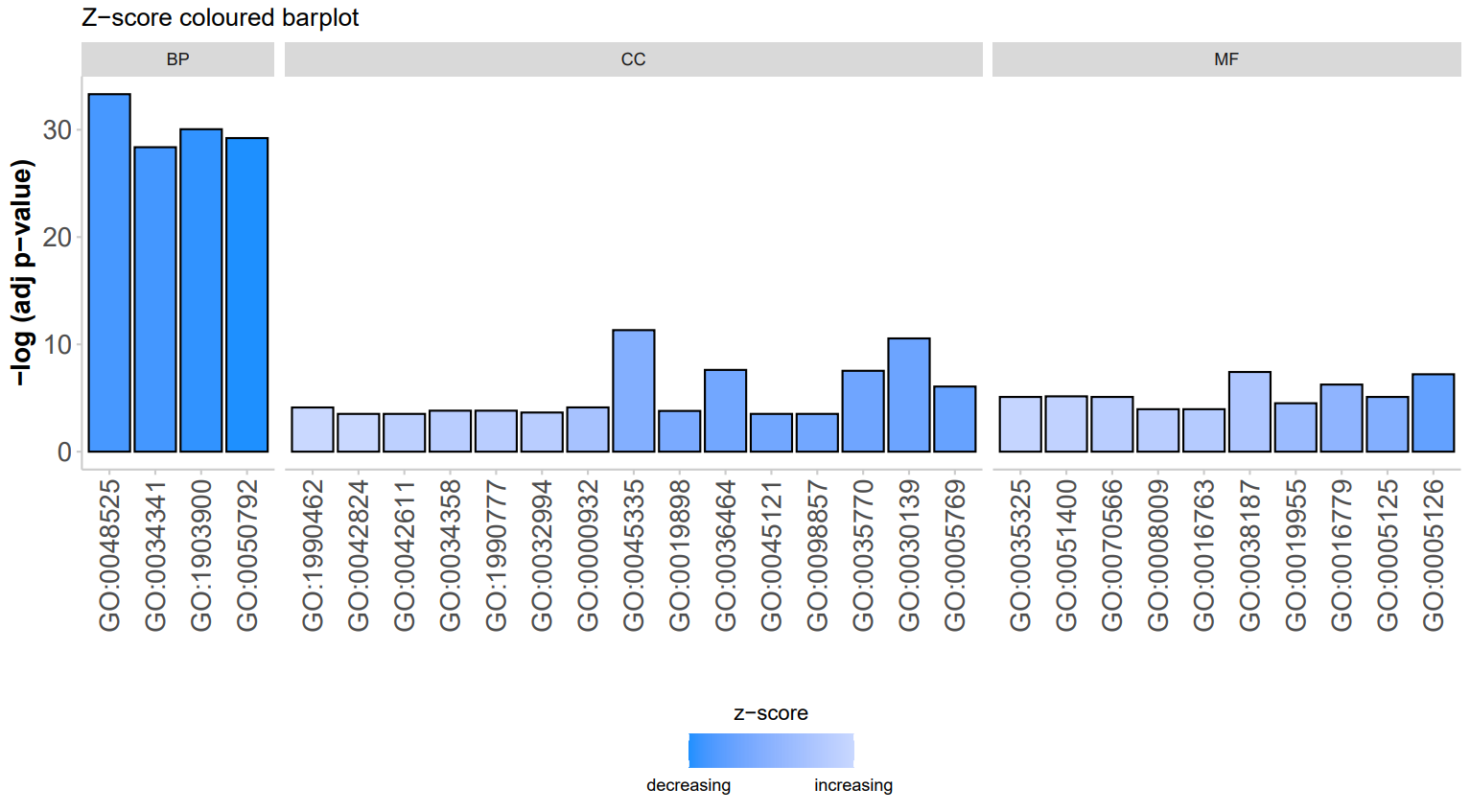

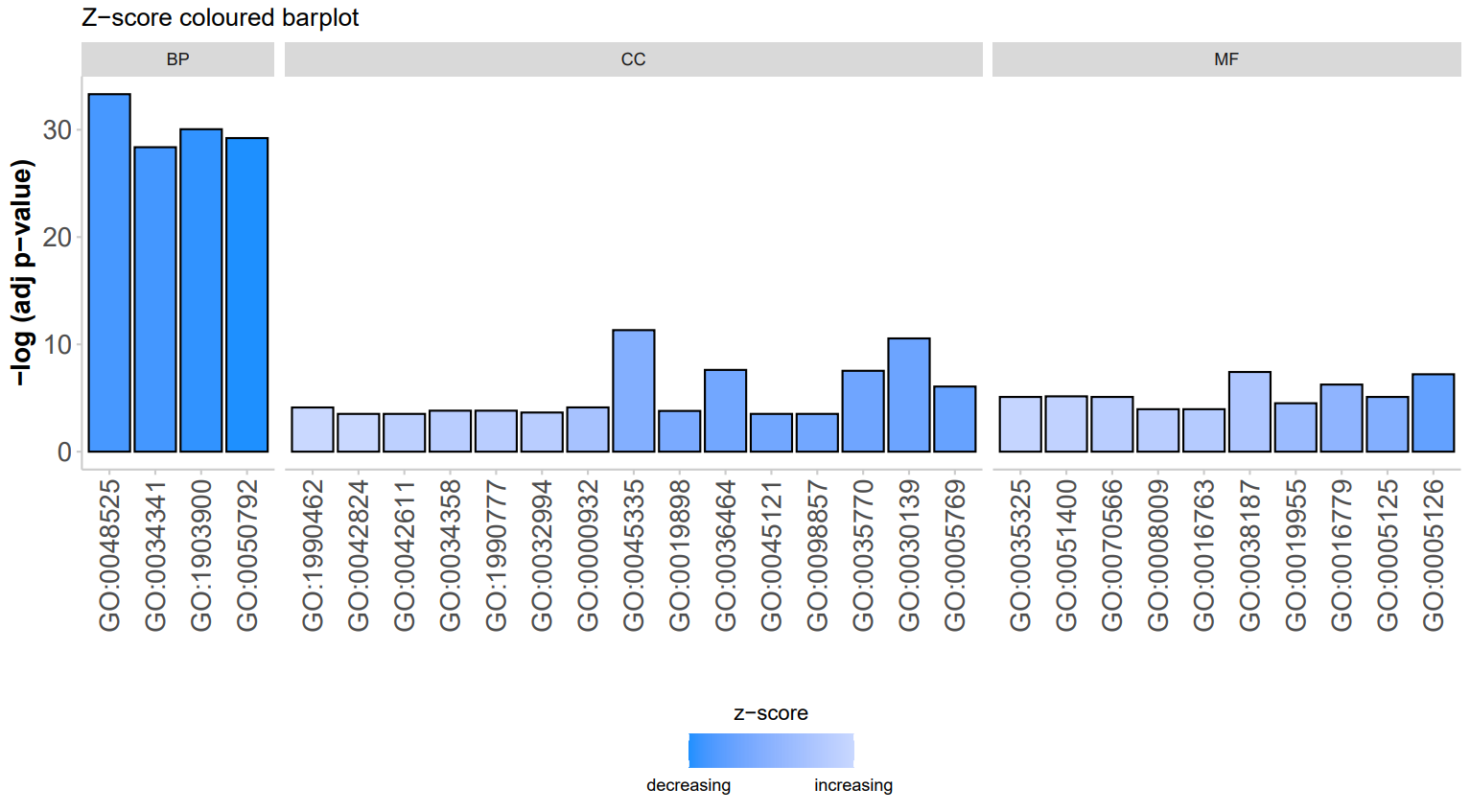


**(A)**

**(B)**

**GO (Up-regulation)**

**GO (Down-regulation)**

**Figure S1. GO Enrichment Analysis of Macrophages Stimulated with LPS and Treated with ESS.**

We conducted a comprehensive GO enrichment analysis to explore gene regulation's functional implications in macrophages following LPS stimulation and ESS treatment. Our analysis encompasses biological processes, molecular functions, and cellular components, visually depicted in a Z-score colored barplot. This representation reveals the degree of (A) upregulation and (B) downregulation within these categories.

**Supplementary Figure 2.**


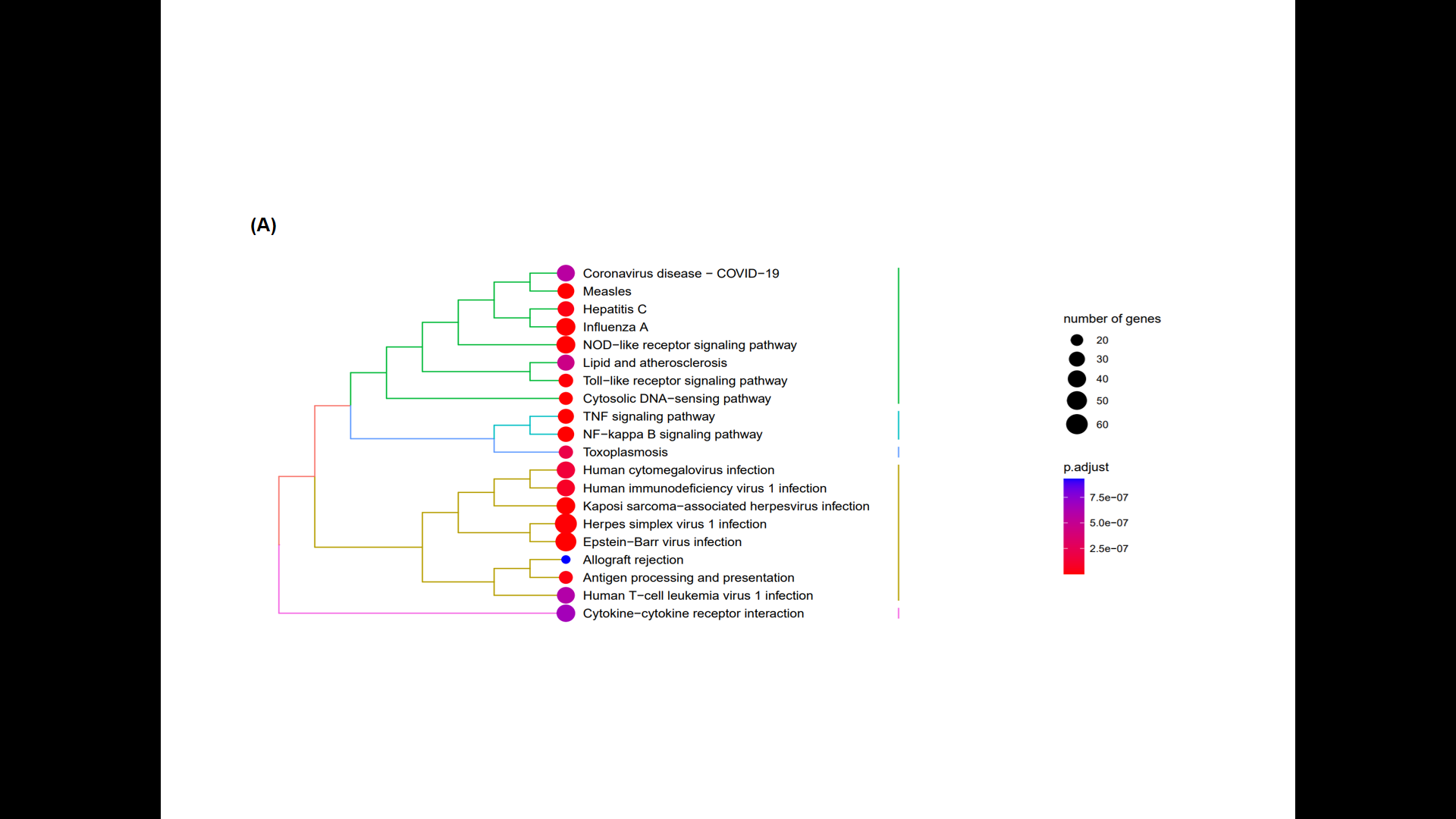


**Figure S2. KEGG Pathway Enrichment Analysis of Differentially Expressed Genes in Macrophages Stimulated with LPS and Treated with ESS**. Differential gene expression (DEG) analysis classified genes into up-regulated and down-regulated categories, followed by KEGG pathway analysis. (A) KEGG pathway maps visualize the down-regulated genes. The data analysis employed a threshold of |log2 fold change| ≥ 0.58 and FDR < 0.05.

**Supplementary Figure 3.**


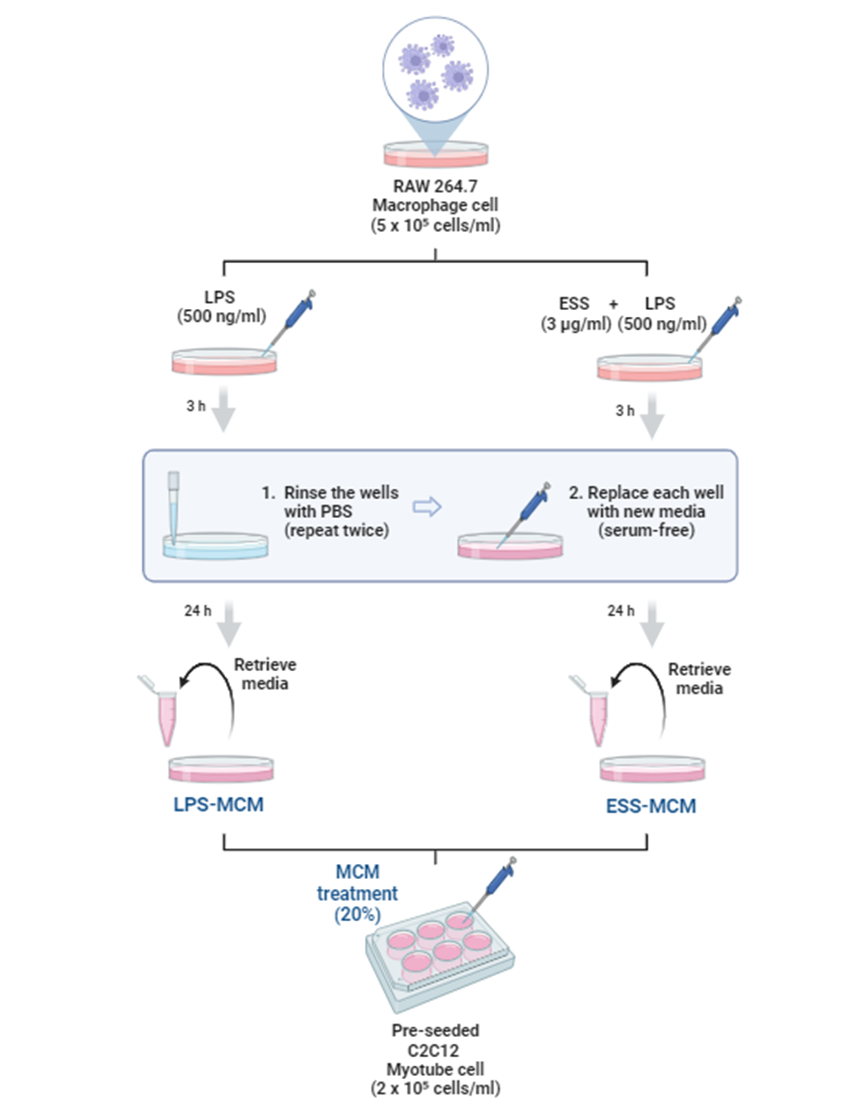


**Figure S3. Process for collecting macrophage-conditioned media(MCM).**

**Supplementary Figure 4.**

**
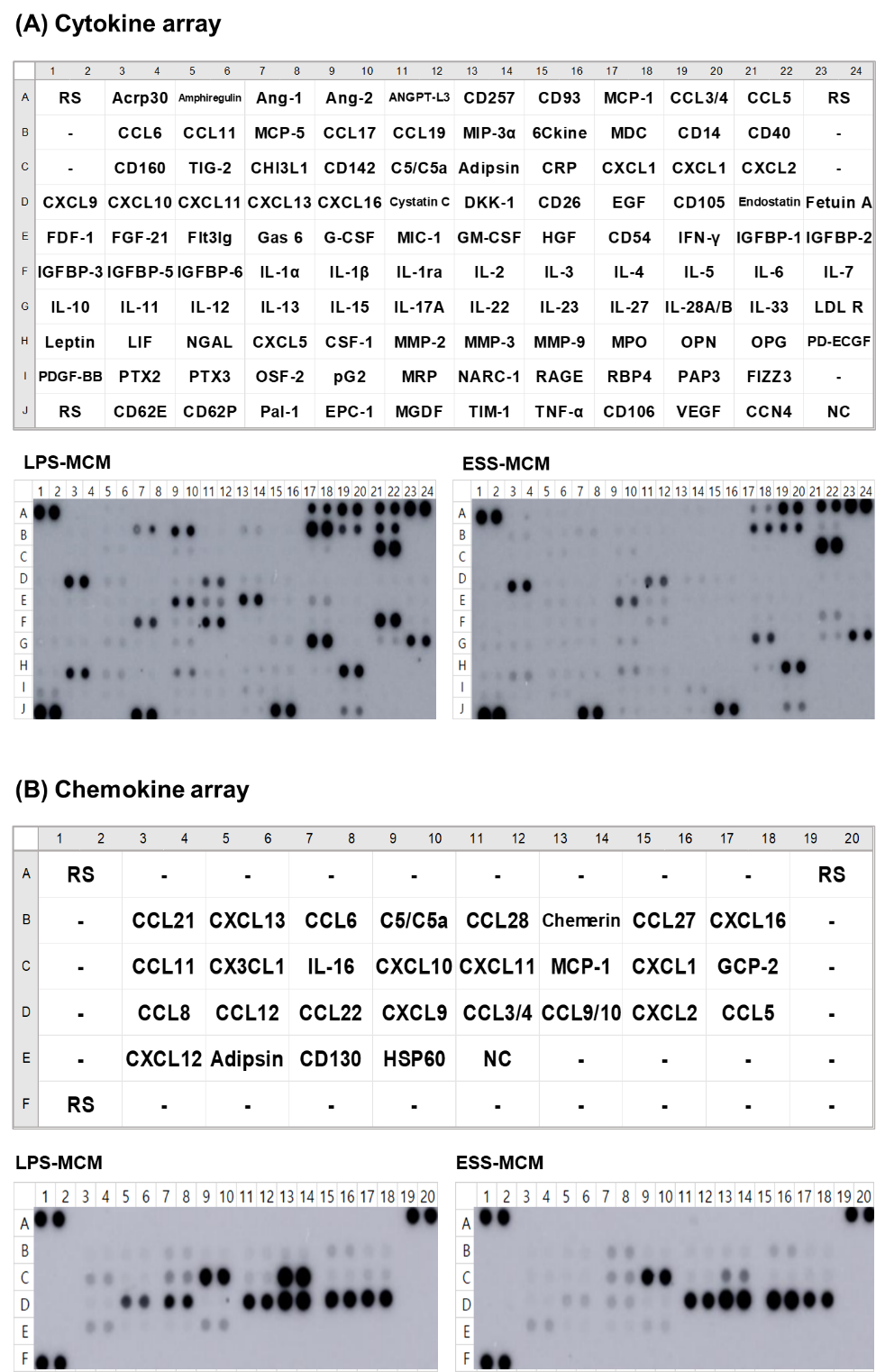
**

**Figure S4. Representative Blots of Cytokine and Chemokine Arrays.** This figure displays representative blots from the cytokine (A) and chemokine (B) array analyses of LPS-MCM and ESS-MCM using the Proteome Profiler array kit.

**Supplementary Table 1.**

| **Gene** | **Forward** | **Reverse** |
| --- | --- | --- |
| **MAFbx** | ATG CAC ACT GGT GCA GAG AG | TGT AAG CAC ACA GGC AGG TC |
| **MuRF1** | TGT CTG GAG GTC GTT TCC G | GTG CCG GTC CAT GAT CAC TT |
| **Trim32** | GAA CGC CGC ATC CAG GAA | GGA CTT CTC AAC CTC AGC CAA G |
| **KLHL40** | CCC AAG AAC CAT GTC AGT CTG GTG AC | TCA GAG TCC AAG TGG TCA AAC TGC AG |
| **KLHL41** | GAA GAT GGT CTT TCA GCT TCA GTT | AGT GGG TGC AAA CTC TTT AGA TTC |
| **Cblb** | GAG CCT CGC AGG ACT ATG AC | CTG GCC ACT TCC ACG TTA TT |
| **Foxo1** | AGT GGA TGG TGA AGA GCG TG | GAA GGG ACA GAT TGT GGC GA |
| **Myostatin** | CTC ATC GCA GTC AAG CCC AA | GAG AAG ATG GGC TGA ATC CC |
| **INOS** | CAC CTT GGA GTT CAC CCA GT | ACC ACT CGT ACT TGG GAT GC |
| **COX-2** | ATC TGG CTT CGG GAG CAC AA | GTG GTA ACC GCT CAG GTG TT |
| **IL6** | CCC ACC AAG AAC GAT AGT CA | CTC CGA CTT GTG AAG TGG TA |
| **IL18** | CAG GCC TGA CAT CTT CTG CAA | TCT GAC ATG GCA GCC ATT GT |
| **IL27** | TCG ATT GCC AGG AGT GAA CC | AAG TGT GGT AGC GAG GAA GC |
| **TNF-α** | TGG AAC TGG CAG AAG AGG | AGA CAG AAG AGC GTG GTG |
| **IFN-β** | TCA AGT GGC ATA GAT GTG GAA GAA | TGG CTC TGC AGG ATT TTC ATG |
| **LIF** | GCC AAT GCT CTC TTC ATT TCC TAT TAC A | CCA TGG AAA GAT GGG AAG TCT GT |
| **CCL2** | TCA GCC AGA TGC AGT TAA CGC | TCT GGA CCC ATT CCT TCT TGG |
| **CCL4** | GAA ACA GCA GGA AGT GGG AG | CAT GAA GCT CTG CGT GTC TG |
| **CXCL2** | TCC AGG TCA GTT AGC CTT GC | CGG TCA AAA AGT TTG CCT TG |
| **CXCL3** | GAT ACT GAA GAG CGG CAA GTC | AAG ACA CAT CCA GAC ACC GTT |
| **CXCL10** | AGT AAC CCA AGT GCT GCC GTC | CTC CAG TTA AGG AGC CCT TTT AG |
| **CXCL11** | ATG AAC AGG AAG GTC ACA GC | GAT GTC ACA TGT TTT GAC GC |
| **ICAM-1** | TCC TAA AAT GAC CTG CAG ACG | AGT TTT ATG GCC TCC TCC TGA |
| **CD40** | GCT ATG GGG CTG CTT GTT GA | ATG GGT GGC ATT GGG TCT TC |

**Table S1. Primers for RT-qPCR amplification of gene expression**
